# Correlation between microRNA-320 and postoperative delirium in patients undergoing tibial fracture internal fixation surgery

**DOI:** 10.1186/s12871-022-01612-w

**Published:** 2022-03-22

**Authors:** Bin Wang, Zeng Yin, Yanan Lin, Xiyuan Deng, Fanghao Liu, He Tao, Rui Dong, Xu Lin, Yanlin Bi

**Affiliations:** 1 Department of Anesthesiology, Qingdao Municipal Hospital Affiliated to Qingdao University, NO. 5 Donghai Middle Road, Qingdao, 266071 Shandong China; 2 Department of Emergency, Qingdao Municipal Hospital Affiliated to Qingdao University, Qingdao, Shandong province China; 3 Department of Anesthesiology, Weifang Medical University, Weifang, Shandong province China; 4 Department of Anesthesiology, Dalian Medical University, Dalian, Liaoning province China; 5 Department of Anesthesiology, Drum Tower Hospital Affiliated to Nanjing University Medical School, Nanjing, China

**Keywords:** MicroRNA-320, Postoperative delirium, IGF-1, APP, Hippocampus, Surgery

## Abstract

**Background:**

Although the incidence of postoperative delirium (POD) in the elderly after surgery are rising as individuals are living longer, the pathogenesis of POD remains poorly understood. It has been suggested that miRNA-320 may play a role in POD based on animal study and human study.

**Methods:**

We first carried out an animal study, and designed and conducted a human study based on the result of animal study. The aged rats were randomly assigned to five groups: the control (C), anesthesia and surgery (AS), saline (NS), agomir-320 (AG), and antagomir-320 (AT) groups. Postoperative spatial learning and memory in rats were analyzed by the Morris water maze and the open field tests. The plasma levels of insulin-like growth factor-1 (IGF-1), amyloid precursor protein (APP) proteins, miRNA320 and IGF-1mRNA were measured by ELISA and qRT-PCR, respectively. A total of 240 Chinese Han patients over 65 years who underwent tibial fracture internal fixation were included in the PNDABLE study. POD cases and non-POD controls (1:1 matched) were selected by an anesthesiologist using Confusion Assessment Method.

**Results:**

For Group AS, the escape latency was significantly longer and the ratio of time spent in the target quadrant was significantly reduced, APP and miR-320 were upregulated and IGF-1mRNA was downregulated compared with Group C. For Group AG, the escape latency was significantly longer and the ratio of time spent in the target quadrant was significantly reduced, APP and miR-320 were upregulated and IGF-1mRNA was downregulated compared with Group AS. For Group AT, the escape latency was significantly reduced and the ratio of time spent in the target quadrant was significantly longer, APP and miR-320 were downregulated and IGF-1mRNAwas upregulated compared with Group AS. Compared with NPOD patients, the expressions of plasma miR-320 and APP protein were increased and the expression of plasma IGF-1 mRNA was decreased in POD patients after surgery.

**Conclusions:**

MiRNA-320 might play a role in up-regulating the levels of IGF-1mRNA and APP protein, which offered a new target for POD treatment.

**Trial registration:**

Correlation of perioperative neurocognitive disorders with lifestyle and biomarkers. ChiCTR2000033439. Registered 1 June 2020.

**Supplementary Information:**

The online version contains supplementary material available at 10.1186/s12871-022-01612-w.

## Background

Postoperative delirium (POD) refers to persistent memory, abstract thinking and disorientation after anesthesia [1]. These neurological complications are characterized by disturbances of memory, attention, consciousness and information processing, and sleep-wake cycle disorders [2, 3]. A clinical study showed that 25.8% of patients developed POD7 days after surgery [4]. However, the proportion of aged patients who need operations is growing year by year, and the incidence rate of postoperative POD increases accordingly. Despite several hypotheses such as neuroinflammation [5], increased amyloid-beta (Aβ) [6], microglia activation and neuronal apoptosis [7], the pathogenesis of POD remains to be determined. Thus, elucidating the underlying molecular mechanisms of POD is crucial for its prevention, diagnosis and treatment.

MicroRNAs (miRNAs) comprise a group of endogenous, highly conserved, non-coding single-stranded small molecules containing 21–23 bases, which can regulate target genes through the degradation of mRNAs or post-transcriptional inhibition [8]. MiRNAs are also involved in the development of neurodegenerative diseases such as neuromuscular failures, frontotemporal dementia [9], cerebral ischemic stroke [10] and Alzheimer’s disease (AD )[11]. A growing body of evidence have showed that miRNAs play an essential role in the development of POD by negatively regulating the expressions of downstream target proteins [12]. For example, increased miRNA-572 can bind to the 3’-untranslated region (3’UTR) region of neural cell adhesion molecule 1 (NCAM1) to downregulate the expression of NCAM1, thereby protecting neurons from degeneration and necrosis, and improving early postoperative cognitive dysfunction [13]. Previous studies have shown that miRNA-320 may be involved in the regulation of cerebral and myocardial I/R by inhibiting insulin-like growth factor I (IGF-I )[14]. IGF-1 plays a critical role in regulating body growth and metabolism, and affects multiple cerebral functions. It can promote development, neuronal excitability, myelin sheath production, angiogenesis, synaptogenesis, and neuronal survival, growth and differentiation [15]. In addition, IGF-I stimulates the proliferation and survival of a variety of cell types, and is considered a universal cytoprotective molecule that can protect cells from free radicals and apoptosis [16]. In particular, low plasma IGF-1 level was associated with poor cognitive performance [17, 18]. It also been showed that exogenous IGF-1 can improve declined cognition in aged rats [19] and inhibit Aβ production through α-secretase and β-secretase in the hippocampus [20]. Amyloid precursor protein (APP) can be hydrolyzed in two ways. First, it is degraded by α-secretase in normal physiological conditions. Second, it generates soluble β-APP8 and C99 through β-site APP-cleaving enzyme-1 (BACE-1), and C99 is hydrolyzed by γ-secretase to generate insoluble amyloid-β (Aβ) [21].

To explore the molecular mechanisms underlying POD, we analyzed the expression levels of miRNA320 following surgery by an animal study and a human study, which might provide novel strategies for the prevention and treatment of POD.

## Methods

The animal study was conducted at Qingdao Municipal Hospital Animal Laboratory in China from January 2021 to March 2021. A total of 150 male C57BL/6 rats (age, 12–14 weeks; weight, 20–25 g) were provided by Dong Chuang Animal Science and Technology Service Company (SCXK Xiang 2009-0012) and housed under a 12 h light/dark cycle with free access to water and food. All animals were acclimatized to their new environment at 24–26 °C for 7 days before the commencement of experiments conducted following the guidelines for care and use of laboratory animals recommended by the Animal Ethics Committee of Qingdao Municipal Hospital. This study was reported in accordance with ARRIVE guidelines. The experiments were also conducted based on the standards set forth in the 8^th^ Edition of Guide for the Care and Use of Laboratory Animals published by the National Academy of Sciences, National Academies Press, Washington DC, USA [22]. The study was approved by the clinical trial and the animal ethics committees of Qingdao Municipal Hospital.

A total of 240 adults over the age of 65 who underwent tibial fracture surgery from August 2020 to January 2021at Qingdao Municipal Hospital, Qingdao City, China, were asked to participate in the present study. The protocol was approved by the institutional review board of Qingdao Municipal Hospital Human Research Committee and was registered in Chinese Clinical Trial Registry (ChiCTR2000033439). All methods were performed in accordance with the relevant guidelines. All patients signed the written informed consent.

### Animal Study

#### Tibial fracture internal fixation surgery

A total of 150 rats were randomized into five groups (*n*=30/group): control (C), anesthesia and surgery (AS), saline (NS), agomir-320 (AG), and antagomir-320 (AT) groups. The control group underwent no treatment, while rats in the AS group were given continuous inhalation of 1.5–2% isoflurane for intubation, followed by 1.5% isoflurane and mechanical ventilation with 100% oxygen for anesthesia maintenance; then, the rats underwent tibial fracture internal fixation. The rats in the NS group were injected with normal saline, those in the AG group were injected agomir-320, and those in the AT group were injected antagomir-320 into the hippocampus tissues before introducing a tibial fracture.

The surgical model was established based on a previous report [23], and general anesthesia was maintained with 2.0 ± 0.4% isoflurane carried by 100% oxygen at a flow rate of 2 L/min for about 20 min, and supplemental analgesia was given via intraperitoneal injection of buprenorphine (0.3 mg/kg) in saline. A 1.5-cm skin incision was made under the knee, the soft tissues were separated to expose the bone, and the periosteum was stripped for a distance of 10 mm circumferentially. Then, a hole was drilled at the level of the tibial tuberosity to insert a 0.3-mm fixation pin into the intramedullary cavity. After the tibia was fixated internally, the bone was fractured in the mid-diaphysis (tibial, mid-shaft) using a surgical scalpel and scissors, and the skin was closed with 4-0 nylon suture. During this process, the rats were placed on the heated pads to maintain the tactical temperature at 37.0 ± 0.5 °C until they were awakened, and the local lidocaine ointment was applied to the incision site every 8 h for 3 days postoperatively.

The current POD models have been mainly established through splenectomy [24], partial hepatectomy [25], and the tibial fracture internal fixation surgery. Spleen is an immune organ that plays a major role in immune regulation, and splenectomy is less common than other types of surgery in clinic practice. However, the blood loss during partial liver resection was large, and the blood volume of the rat was relatively limited. The tibial fracture internal fixation surgery, similar to clinical abdominal surgery, was often performed to induce the POD model.

#### qRT-PCR analysis

Purified miRNA was reverse-transcribed into cDNA (Tiangen-KR211) on a PCR machine (Bori), and the OD value was determined by an ultraviolet spectrophotometer. The detection primers were *miR320* and *IGF-1,* and those of the reference gene (Tiangen-CD200) were used to prepare the reaction for fluorescence quantitative PCR (Tiangen-FP411), according to the manufacturer’s instructions: *IGF-1* forward primer 5’-AAAAATCAGCAGTCTTCCAACC-3’, reverse primer 5’-CCTGTGGGCTTGTTGAAATAAA-3’; miR-320 5’-CUCCCCUCCGCCUUCUCUUCCCGGUUCUUCCCGGAGUCGGGAAAAGCUGGGUUGAGAGGGCGAAAAAGGAUG-3’. SYRB Green method was used to conduct the PCR reaction on the fluorescent quantitative PCR instrument (Shanghai Hongshi), and the corresponding program was used to perform the amplification and fuse curve analysis. The Tm values of the target and reference genes were recorded for calculation.

#### Intrahippocampal injection

After isoflurane induction and intraperitoneal anesthesia with 3% pentobarbital sodium, the rats were placed on the stereotaxic apparatus, and the head was fixed. After the skin of the head was disinfected, lidocaine was injected subcutaneously, and a 1.5-cm skin incision was made along the middle of the head. For the delivery of agomir-320, antangomir-320, or normal saline, we described the bregma as the zero point by the stereo locator. The hippocampus relative to bregma was 1.8 mm at the anterior/posterior axis, ± 2.5 mm at the lateral/medial axis, and -2.3 mm at the dorsal/ventral axis. Then, a shallow hole was drilled, and the reagents (4 μL, 0.25 nmol/μL) were injected into the hippocampus through a 5 μL syringe at a rate of 0.2 μL/min, with the needle retention time of 5 min, and then were withdrawn slowly.

#### Blood gas assessment

To examine potential secondary effects of isoflurane, particularly hypoxia, hypercapnia and hypoglycemia, we evaluated five rats per treatment group as cardiorespiratory controls. An equivalent of 2 ml blood sample was collected by cardiac puncture upon isoflurane exposure. Arterial blood gas and blood glucose levels were assessed using a portable blood gas analyzer (VetStat Electrolyte and Blood Gas Analyzer).

#### Cognitive assessment via Morris water maze

The MWM system (Shanghai XinRuan Information Technology Co., Ltd.) comprised an imaging device (for swimming tracking) and a circular test pool 120 cm in diameter and 40 cm in height) with a cylindrical platform (diameter 10 cm and height 30 cm) located 2 cm below the water surface. For the swimming test, rats were acclimated to the platform for 30 s before entering the water from four different quadrants on the pool wall. The time spent before finding the platform (escape latency) was recorded to assess the learning and memory abilities of the rats before surgery. Rats that could not find the platform within 60 s were guided to it and allowed 10 s to acclimate. The swimming test was carried out for 5 consecutive days, 4 trials/day, starting from 1-week before surgery. In the spatial test, the platform was removed before placing the rats in the water for 60 s, and the proportion of swimming time in the target quadrant was evaluated. Specific tests were performed one day preceding the operation and 1, 3, and 7day following surgery. Escape latency and percentage of target quadrant were analyzed.

#### Cognitive assessment via open field test (OFT)

The OFT was similar to that described by Rajizadeh et al. [26], and was completed before MWMT to exclude the alteration that occurred in motion, which affected the swimming. The apparatus was a white plastic chamber of 50 × 50 × 40 cm,^3^ which was divided into the center and the peripheral parts. Each rat was placed individually into the center of the area and permitted to freely explore for 5 min, and all the movements were automatically recorded by a digital camera placed above this area. The noise was avoided during the experiment, and at the end of the testing, the area was cleaned with 75% alcohol to avoid the presence of olfactory cues. Finally, the parameters of general locomotion distance, time spent in the center area, rearing times, and the high-leaning behavior were recorded to evaluate the activity and the anxiety of the rats one day before surgery and on 1-, 3-, and 7-day following surgery.

After an assessment of cognitive function, ten rats from each of the five groups were exposed to a high concentration of isoflurane, followed by cervical dislocation to sacrifice the rats and harvest the hippocampus. The five hippocampal samples were evaluated by Western blot. The remaining five hippocampal samples were assessed by qRT-PCR.

#### Western blot analysis

The hippocampus tissues were lysed with lysis buffer containing protease inhibitor (Biyuntian Biotechnology Research Institute) and homogenized with homogenizing pestles on ice. The supernatant was obtained by centrifugation at 4 °C for 5 min, and the protein concentration was estimated using BCA. The protein was separated by polyacrylamide gel electrophoresis and transferred to the PVDF membrane. Subsequently, the membrane was blocked with 5% skim milk powder in TBST for 2 h at room temperature and probed with IGF-1 and APP antibodies (1:1000, Abcam, UK) overnight at 4 °C, followed by incubation with horseradish peroxidase-labeled secondary antibody (1:5000, Beijing Zhongshang Jinqiao Technology Co., Ltd) for 2 h at room temperature. The β-actin served as the internal control. The image was acquired and analyzed using Image J software (NIH, USA) to determine the target protein expression level based on the ratio of the gray value of the target protein band to that of the internal reference β-actin.

#### qRT-PCR Analysis

After the water maze test 1, 3, and 7 days following surgery, 5 rats were randomly selected and given a high concentration of inhaled anesthesia. After the righting reflex disappeared, the rats were decapitated and sacrificed. The skin at the top of the skull was dissected, and the parietal bone and occipital bone were removed. Then, the brains were removed, and the tissues were placed on ice. According to the anatomical map, the mouse hippocampus was extracted, and the hippocampus tissue was placed in an RNA preservation solution (Biyuntian Biological Reagent Research Institute) at 4 °C overnight. The RNA preservation solution was discarded on the following day, and the hippocampus tissue was preserved at -80 °C. Total RNA and miRNA from the hippocampus were extracted with an ultra-pure RNA extraction kit (Tiangen Biochemical). The enhanced miRNA cDNA first-strand synthesis kit and FastKing cDNA first-strand synthesis kit (Tiangen Biochemical) were used to reversely transcribe the corresponding RNA into cDNA, which was detected using the SuperReal fluorescence quantitative premix kit and minute enhanced miRNA fluorescent quantitative detection kit (Tiangen Biochemical) on a real-time quantitative PCR system. The *β-tubulin* and *U6* served as internal controls. The primers used included: *IGF-1*, forward primer 5’-GAGACGCTCTTCAGTTCGTGTG-3’, reverse primer AGCTTCCTTTTCTTGTGTGT; *miR-320*, 5’-GCTGGGTTGAGAGGGCGA-3’; *U6*, a primer: 5’-CGCAAGGATGACACGCAAATTC-3’. After *β-tubulin* and *U6* were normalized, the expression of the target gene was measured using 2-^ΔΔct^. The dissolution curve was measured immediately after amplification showed a single peak, indicating product specificity.

We first carried out an animal study, and designed and conducted a human study based on the result of animal study.

#### Human Study from the PNDABLE study

##### PNDABLE study

The Perioperative Neurocognitive Disorder and Biomarker Lifestyle (PNDABLE) study explored the pathogenesis, risk factors, and biomarkers of perioperative neurocognitive disorders in northern Chinese Han population. This study also aimed to identify lifestyle factors that might affect the risk of POD in a non-demented northern Chinese Han population, providing a basis for disease prevention and early diagnosis [27].

A total of 240 Chinese Han patients with normal cognition who underwent tibial fracture internal fixation (no gender limitations) at Qingdao Municipal Hospital from August 2020 to January 2021 were enrolled in the PNDABLE study. The patients were 65–90 years, weighing 50–80 kg, with American Society of Anesthesiologist (ASA) physical status I-II, and received combined epidural anesthesia. The patients were divided into two groups: POD and non-POD (NPOD) group. POD cases and non-POD controls were frequency-matched (1:1). Specifically, one non-delirium control was randomly selected for each POD case from the source population based on five matching variables, including age, diagnosis, ASA physical status, duration of surgery and intraoperative blood loss. These variables were listed in the European Society of Anesthesiology evidence-based and consensus-based guidelines for the prevention and treatment of POD and were considered risk factors for POD after tibial fracture internal fixation. The exclusion criteria were as follows: 1) preoperative Mini-Mental State Examination (MMSE) score < 24 points; 2) a history of neurological and mental diseases, such as AD, Parkinson’s disease (PD), and cerebrovascular accident; 3) drug or psychotropic substance abuse, as well as long-term use of steroid drugs and hormone drugs; 4) preoperative III-IV hepatic encephalopathy; 5) recent major surgery; 6) severe visual and hearing impairments; and 7) abnormal coagulation function before surgery.

The participants did not receive preoperative medications and were instructed not to drink for 6 h and not to eat for 8 h before surgery. After entering the operating room, ECG, SpO_2_, NBP and opened vein access were routinely monitored. All patients underwent combined spinal-epidural block, with the puncture point between the lumbar 3-4 spinous processes (L_3-4_). After successful puncture, 2–2.5 mL of ropivacaine (0.66%) was injected at the site for about 30 s. After anesthesia, the sensory level was controlled below the T_8_ disc. During the surgery, oxygen was inhaled via a mask at 5 L/min to maintain blood pressure within ± 80% of the baseline value. If intraoperative NBP was < 90 mmHg (1 mmHg = 0.133 kPa) or the value decreased by > 20% of baseline, ephedrine (5 mg) was injected intravenously. If heart rate (HR) was < 50 beats/min, atropine (0.5 mg) was injected intravenously. In addition, patient-controlled intravenous analgesia (butorphanol 0.1 mg/mL + tropisetron 0.05 mg/mL, diluted with normal saline to a total volume of 100 mL) was administered for acute postoperative pain management. After the operation, the patient was taken to an anesthesia resuscitation room (PACU). If no abnormalities were detected during the 30-min observation period, the patient could return to the low-flow oxygen ward with continuous monitoring of vital signs.

We interviewed all patients the day before surgery and collected their baseline data, including age, sex, body mass index (BMI), ASA physical status, and education, as well as MMSE, Confusion Assessment Method (CAM) and Memorial Delirium Assessment Scale (MDAS) scores. Cognitive assessment related to dementia was conducted by neurologists.

##### Neuropsychological tests

MMSE scoring was completed by neurologists 1 day before surgery to assess preoperative cognitive status and record relevant medical history. Patients with MMSE score < 23 points were excluded. All participants underwent interviews preoperatively.

Delirium assessments were performed by an anesthesiologist from 9:00-11:00 and 17:00-19:00 1–7 days after surgery (or before discharge). Next, we assessed pain using the visual analog scale (VAS) scored from 0–10, with lower scores indicating less pain. POD was defined by CAM, and POD severity was measured using the MDAS score. CAM-positive and MDA-positive patients were monitored postoperatively.

##### Enzyme-linked immunosorbent assay (ELISA)

Before anesthesia and 1 day after surgery, 3ml of venous blood was extracted and placed into the EDTA anticoagulant tube. Plasma was thawed from -80 °C to room temperature. A volume of 50 μL standard products was added to the standard wells at different concentrations and 50 μL diluent was added to the blank wells. A volume of 50 μL was added to the sample wells, followed by an addition of 50 μL test solution A (1:100 dilution). The plate was mixed with gentle shaking, covered with plastic film, and incubated at 37 °C for 1 h. Subsequently, each well was washed with 350 μL washing buffer (1:30) for about three times, 100ul of the working solution B was added, and the plate was covered with plastic film and incubated at 37 °C for 30 min. Then 50 μL termination solution was added to each well to stop the reaction, and the blue complex turned to yellow. The absorbance was measured at 450 nm, and the concentrations of IGF-1 and APP were calculated according to the standard curve regression equation.

### Statistical analysis

The characteristics of patients considered as potential covariates included age and education. We controlled for age and education due to their known effects on cognitive status [28]. The preliminary test in this study found that 2 covariates (age and education) were expected to enter the Logistic regression. The POD incidence was 10%, and the loss of follow-up rate was assumed to be 20%, so the sample size was calculated to be 240 cases.

Kolmogorov–Smirnov test was used to determine whether the data were normally distributed. In the case of non-normal distribution, Mann–Whitney U test was used for data analysis. Normally distributed data were expressed as mean ± standard deviation (SD). Two independent-samples t-test for the analysis of the expression of the miR-320, IGF-1mRNA, IGF-1, and APP were used for data analysis in patients. One-way analysis of variance (ANOVA) was used to analyze the learning and memory abilities, and the expressions of miR-320, *IGF-1* mRNA, IGF-1, and APP proteins in the hippocampus of the rats.

SPSS 20.0 (SPSS, USA) was used for statistical analyses. *P* < 0.05 indicated a significant difference.

## Results

### Expressions of miR-320 and APP protein were increased and expressions of the *IGF-1* mRNA was decreased in the plasma of POD patients after surgery

**Fig. 1:**
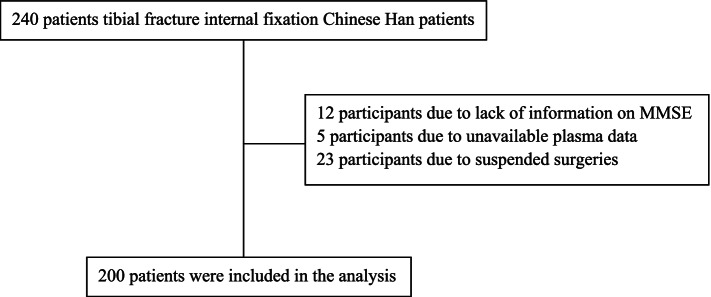
Flow diagram showed selection of eligible patients and the enrollment process

We excluded 12 participants due to lack of information on MMSE, 5 participants due to unavailable plasma data, and 23 participants due to suspended surgeries. Finally, 200 participants were included in this analysis (Fig. 1). The incidence of POD was 11% (*n* = 22) as assessed postoperatively. The demographic and clinical data of the participants were summarized (Table 1).

**Table 1:** Comparison of general condition and surgical condition of tibial fracture internal fixation surgery

Variable	POD(*N* = 22)	NPOD(*N* =22)	*P*-values
Age (year), mean ± SD	75.18±3.53	73.77±4.26	0.239
Sex (female/male)	10/12	11/11	0.763
Body mass index (kg.m^-2^ ), mean ± SD	25.82±3.61	26.74±4.06	0.434
Education level (year), median and 25–75 percentile	9 (7.75-13.25)	11 (10-13)	0.114
ASA physical status (I/II)	16/6	12/10	0.610
Preoperative MMSE scores , median and 25–75 percentile	27 (25-28)	27 (25-28)	0.321
Duration of anesthesia (min) , mean ± SD	141.36±12.45	146.36±14.97	0.235
Duration of surgery (min) , mean ± SD	122.5±11.52	126.14±11.12	0.293
Intraoperative blood loss (ml) , mean ± SD	123.41±12.38	117.73±10.77	0.112
Postoperative the highest MDAS (score) , mean ± SD	20.32±5.63	9.59±4.23	<0.001
Postoperative the highest VAS (score) , median and 25–75 percentile,	2 (1-3)	3 (2-4)	0.413

*Abbreviations*: *POD* Postoperative delirium, *MMSE* Mini-mental state examination, *ASA* American Society of Anesthesiologists, *MDAS* Memorial delirium assessment scale, *VAS* Visual Analogue Scale/Score, *SD* Standard deviation

**Fig. 2:**
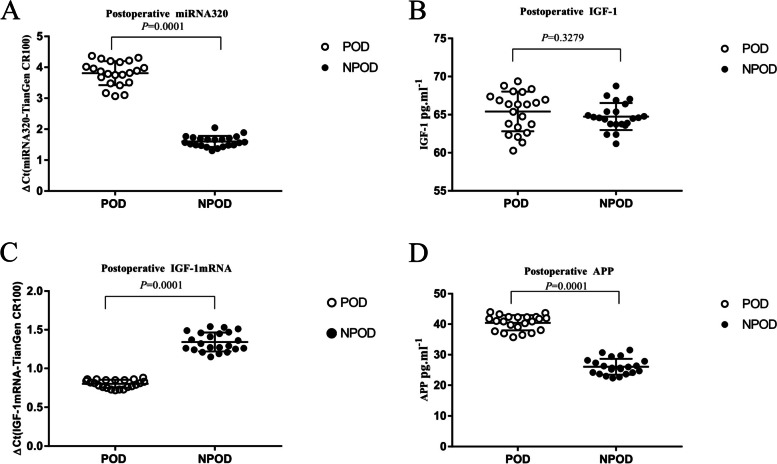
The postoperative plasma concentrations of miR-320 and APP increased and IGF-1 mRNA decreased in POD patients compared with NPOD patients (**A**-**D**) (*P*<0.05)

Compared with NPOD patients, the expressions of the miR-320 and APP protein were increased and the expression of the IGF-1 mRNA was decreased in the plasma of POD patients on 1 day after surgery, but the expression of the IGF-1 protein was not significantly different between NPOD patients and POD patients (Fig. 2).

### Physiological parameters after isoflurane administration in rats

As shown in Table 2, the components of the arterial blood gas tests and blood glucose amounts did not differ significantly among the controls and the other four groups immediately after receiving anesthesia. These data indicated that neither hypoxia nor hypoglycemia occurred in the test rats, and any POD identified after surgery was not related to hypoxia or hypoglycemia.

**Table 2:** Physiological Parameters in the Controls and the Other Test Groups Immediately After Isoflurane Administration

Test Group	pH	PaCO2 (mm Hg)	PaO2 (mm Hg)	SaO2 (%)	Glucose (mmol/L)	Hb (g/dL)
C	7.34 ± 0.08	39.8± 2.6	178 ± 7	96 ± 3	4.8 ± 0.7	136 ± 13
AS	7.34 ± 0.07	40.2 ± 3.1	168 ± 8	97 ± 2	5.2 ± 0.7	137 ± 14
NS	7.33 ± 0.06	39.9 ± 2.8	165 ±9	98± 1	5.3± 0.8	140 ± 12
AG	7.34 ± 0.08	38.6 ± 2.5	164 ± 8	97 ± 2	5.1 ± 0.6	141 ± 13
AT	7.35 ± 0.06	38.6± 2.9	163 ± 8	96 ± 3	5.2 ± 0.7	143 ± 12
*P*	> 0.05	> 0.05	> 0.05	> 0.05	> 0.05	> 0.05

### Impaired learning and memory in rats after surgery

**Fig. 3:**
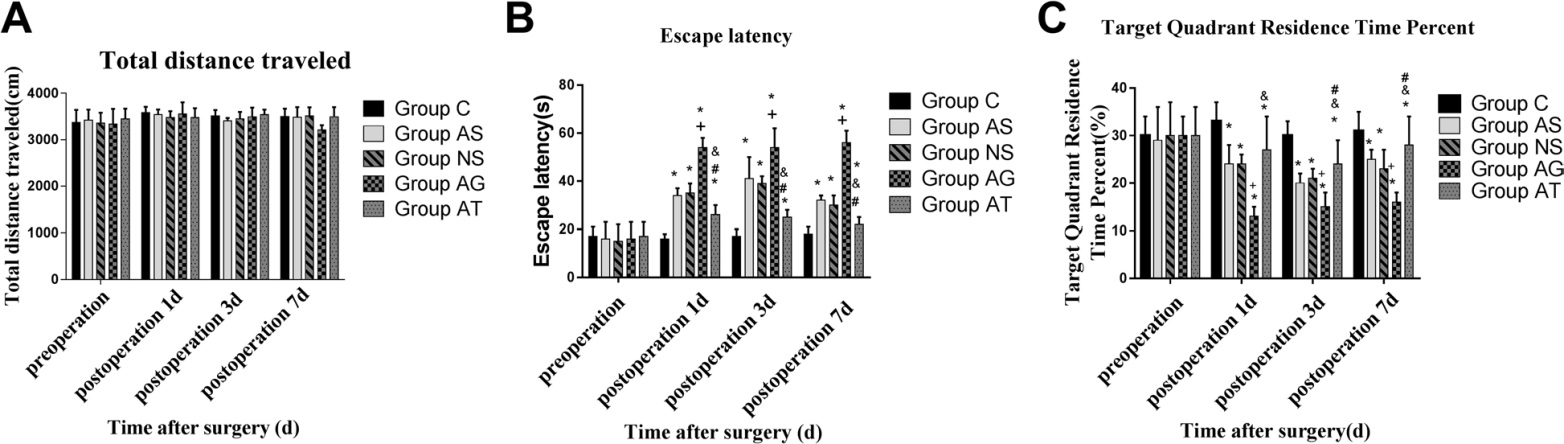
Open Field Test. Compared with the group C, there was no significant statistical difference in the total exercise distance of the rat on the 1^st^, 3^rd^ and 7^th^ day following surgery (**A**)(*P*> 0.05). The morris water maze (MWM) . The escape latency (**B**) was higher and the ratio of time spent in the target quadrant (C) was lower in Group AS, NS, AG and AT, indicating that cognitive function (ie, spatial learning and memory) decreased in aged rats after surgery (all *P* <0.05). The escape latency (**B**) was higher and the ratio of time spent in the target quadrant (**C**) was lower in the Group AG than those in Group AS (both *P*<0.05). The escape latency (**B**) was lower and the ratio of time spent in the target quadrant (**C**) was higher in the Group AT than those in Group AS (both *P* <0.05). The escape latency (**B**) was higher and the ratio of time spent in the target quadrant (**C**) was lower in the Group AT than in Group AG (both *P*<0.05), indicating that the antagomir-320 alleviated this impairment. *Significant at *P*≤0.05 for Groups AS,NS,AG,AT versus Groups C , +Significant at *P*≤0.05 for Group AG versus Group AS. ^#^Significant at *P*≤0.05 for Groups AT versus Groups AS ,^&^Significant at *P*≤0.05 for Groups AT versus Groups AG

We then determined the escape latency and the ratio of time spent in the target quadrant after surgery by MWM and OFT. The result showed that the escape latency in the AS, NS, AG, and AT groups was significantly longer, and the ratio of time spent in the target quadrant was significantly reduced 1, 3 and 7 days after surgery compared to group C. The above findings suggested that surgery aggravated cognitive impairment. The escape latency in group AG was significantly longer, and the ratio of time spent in the target quadrant was significantly reduced 1, 3 and 7 days following surgery compared to group AS. The escape latency in group AT was significantly reduced, and the ratio of time spent in the target quadrant was significantly longer 1, 3 and 7 days following surgery compared to Group AS. The escape latency in group AT was significantly reduced, and the ratio of time spent in the target quadrant was significantly longer 1, 3 and 7 days following surgery compared to Group AG, while no significant difference was noted in the total exercise distance of the rat between each group before surgery (Fig. 3).

### Expressions of miR-320 and APP protein were increased and expressions of *IGF-1* mRNA was decreased in the hippocampus of rats after surgery

**Fig. 4:**
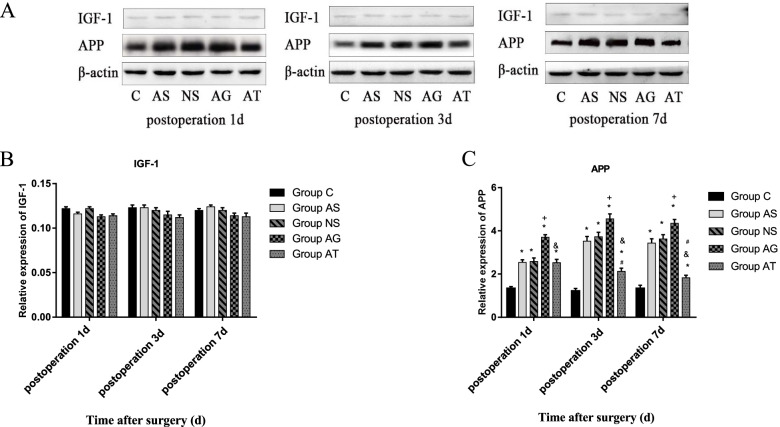
Surgery could upregulate APP protein in hippocampal samples from animals on the 1^st^, 3^rd^ and 7^th^ day following surgery (**A**-**C**). The agomir-320 could upregulate APP protein in the hippocampus of aged rats on the 1^st^, 3^rd^ and 7^th^ day following surgery. The injection with antagomir-320 following splenectomy could deregulate APP protein in the hippocampus of aged rats on the 1^st^, 3^rd^ and 7^th^ day following surgery. There was no significant differences in the expression levels of IGF-1 in hippocampal samples on the 1st, 3rd and 7th day following surgery. *Significant at *P*≤0.05 for Groups AS, NS, AG, AT versus Groups C , †Significant at *P*≤0.05 for Group AG versus Group AS. ^#^Significant at *P*≤0.05 for Groups AT versus Groups AS ,^&^Significant at *P*≤0.05 for Groups AT versus Groups AG

**Fig. 5:**
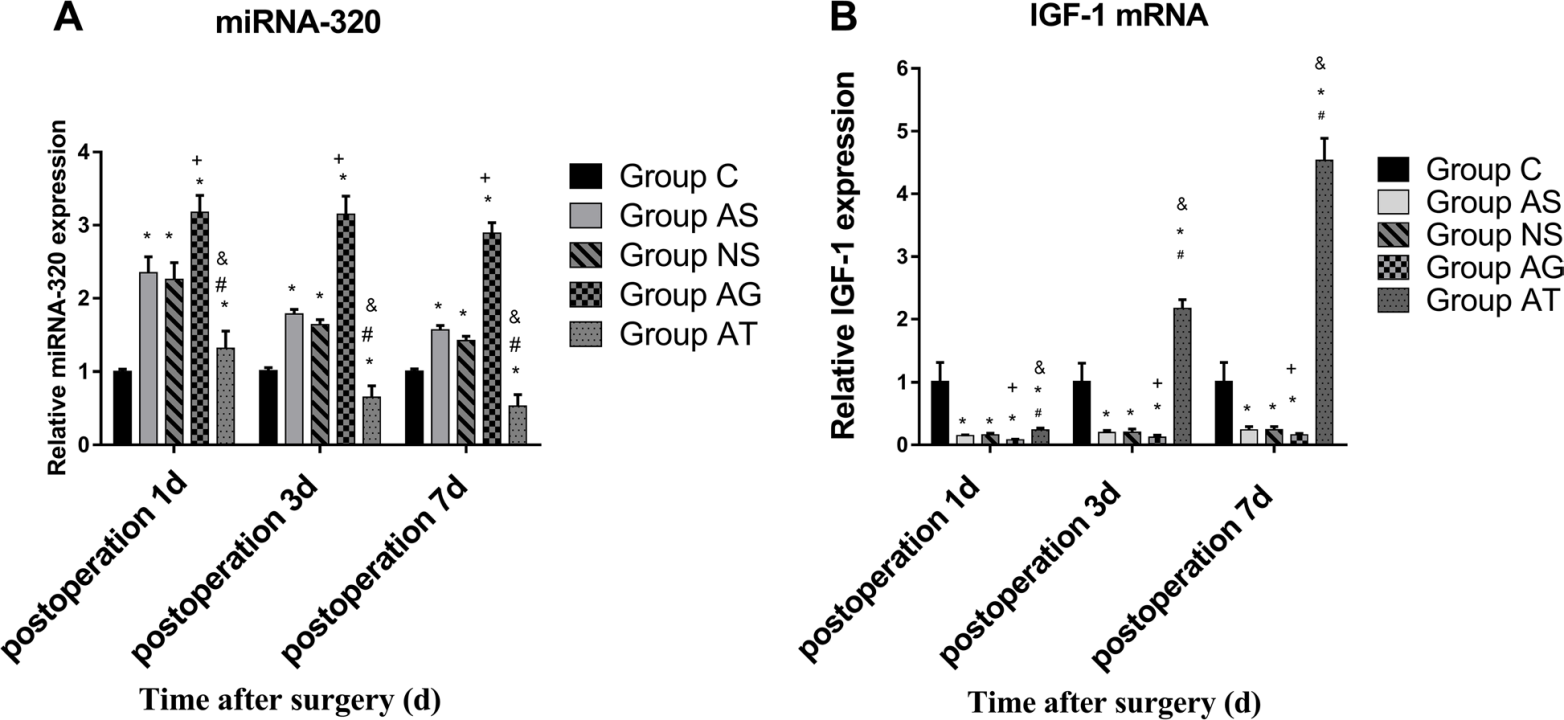
Surgery could upregulated miR-320 and downregulated IGF-1mRNA in hippocampal samples from animals on the 1^st^, 3^rd^ and 7^th^ day following surgery(**A**-**B**). The agomir-320 could upregulated miR-320 and downregulated IGF-1 mRNA in the hippocampus of aged rats on the 1^st^, 3^rd^ and 7^th^ day following surgery. The injection with antagomir-320 following splenectomy could downregulated miR-320 and deregulated IGF-1 mRNA in the hippocampus of aged rats on the 1^st^, 3^rd^ and 7^th^ day following surgery. *Significant at *P*≤0.05 for Groups AS versus Groups C , †Significant at *P*≤0.05 for Group AG versus Group AS. ^#^Significant at *P*≤0.05 for Groups AT versus Groups AS ,^&^Significant at *P*≤0.05 for Groups AT versus Groups AG

We then determined the expression levels of miR-320, IGF-1 mRNA, IGF-1 and APP proteins in hippocampus of rats by qRT-PCR and western Blot. The results showed that the tibial fracture internal fixation surgery significantly upregulated miR-320 and APP protein and downregulated IGF-1 mRNA in hippocampal samples from the rats 1, 3, and 7 days following surgery compared to the control groups. After surgery, agomir-320 upregulated miR-320 and APP protein, and downregulated IGF-1 mRNA in the hippocampus of aged rats compared to the AS and NS groups. On the other hand, antagomir-320 downregulated miR-320 and APP protein, and upregulated IGF-1 mRNA in the hippocampus of aged rats 3 and 7 days following surgery compared to the AS and NS groups. But the expression of the IGF-1 protein was not significantly altered after surgery, injection of agomir-320 and injection of antagomir-320 (Figs. 4 and 5).

## Discussion

In this study, the expression of the miR-320 was increased in the plasma of for POD patients after surgery. The current animal models showed that the expression of the miR-320 was increased after surgery in rats. These results indicated that the occurrence of POD might be related to the changes in miR-320, which might be a critical convergence point in cell signaling.

The miRNAs are highly conserved endogenous RNA transcripts that are cut in the stem ring region [29]. They are single-stranded non-coding RNAs consisting of 21–23 nucleotides. Interestingly, miRNA can bind to the 3’-UTR of the downstream target proteins and degrade mRNA or prevent protein translation, so miRNA plays a negative regulatory role at the post-transcriptional level. Since the discovery of the first miRNA, 1917 human miRNA species have been recorded in the miRBase database by 2019 [30]. The miRNAs constitute the complex interaction networks and organizations, and are characterized by temporal expression specificity, lending an extra identification feature. Furthermore, brain tissue comprises about 70% of the accredited miRNAs that participate in a variety of cellular events such as the regulation of neurons and glial cells, the development of brain tissue and brain, and the process of aging [31]. Some studies have showed that among 269 hippocampus miRNAs, 80 are differentially expressed between young and old rats, and miRNAs participate in brain aging via various mechanisms including synaptic plasticity and cognition, inflammation, neuroprotection, lipid metabolism, and mitochondrial function regulation [32]. The miRNAs are involved in the pathogenesis of central cognition-related diseases, such as PD, frontotemporal dementia [33], ischemic encephalopathy with cognitive changes [34], AD [35], and POCD [36].

OFT can assess the anxiety and autonomic motor abilities of animals [37]. This test takes advantage of the rodents’ natural fear of predators in the open environment, high vigilance, and dare not to stay for a prolonged period, thus generating anxiety-like behavior and constantly moving and exploring the new environment. In this study, total distance of motion assessed by OFT was used to assess the effect of internal fixation of tibial fractures on the voluntary activity in rats. The experimental results showed that compared to the control group, no statistical difference was noted in the total distance of exercise in other groups, indicating that fracture did not have a significant influence on the autonomous exercise ability. Therefore, the accuracy of the water maze experiment could be guaranteed.

In the present study, we observed that the escape latency was significantly longer, and the ratio of time spent in the target quadrant was significantly reduced after surgery. These findings indicated that the surgery following the tibial fracture induced postoperative delirium in the rats, which was consistent with the findings of previous studies [38].

Herein, we found that the expression of the miR-320 was increased and the expression of the *IGF-1* mRNA was decreased in the plasma of POD patients after surgery. Animal sthdys showed that the expression level of miRNA-320 was upregulated, and the expression level of *IGF-1* mRNA were downregulated in the hippocampal samples from the rats 1, 3 and 7 days following surgery. In order to further determine the role of miRNA-320 in POD, the expression of miRNA-320 was interfered in vivo through intrahippocampal microinjection. The normal saline was first used as a control to rule out the effects of microinjection on the hippocampus. The experimental results showed that after injection with normal saline in the hippocampus, no statistically significant change was detected in the escape latency and the percentage of residence time in the target quadrant in MWMT 1, 3 and 7 days following surgery, and no difference was observed in the expression levels of miRNA-320 and *IGF-1* mRNA in the hippocampus, compared with anesthesia and surgery group. After agomir-320 was injected into the hippocampus, the expression of hippocampal *IGF-1* mRNA was decreased, but was upregulated followingantagomir-320 injection. A negative correlation was established between miRNA-320 and *IGF-1* mRNA. The results of animal studies were consistent with those of clinical studies. These results indicated that tmiRNA-320 eventually led to POD by regulating *IGF-1* mRNA. Furthermore, we used miRanda and TargetS to predict miRNA-320 target genes and analyzed the data to identify the intersection. Reportedly, miRNA-320 could specifically bind the 3’-UTR of *IGF-1* mRNA, and double luciferase reporter gene detection study confirmed this finding [39]. Hence, miRNA-320 inhibited the expression of *IGF-1* mRNA, which was also consistent with the current findings.

In this study, the expression of the IGF-1 protein was not statistically significant in both clinical studies and animal studys. These results indicated miRNA-320 might induce IGF-1 protein modification, but did not induce protein quality change. We also found that the levels of APP protein were upregulated in the hippocampal samples obtained from rats after surgery, which was also consistent with the results of clinical studies. After agomir-320 was injected into the hippocampus, the expression of APP protein was increased but decreased after antagomir-320 was injected, and a positive correlation between miRNA-320 and APP was established. The results of this study showed that miRNA-320 altered the expression of APP protein by regulating *IGF-1* mRNA. When the expression of miRNA320 is up-regulated and the expression level of IGF-1mRNA in the hippocampus is decreased, the activity of glycogen synthesis kinase (GSK) located downstream of MAPK and PI3K /Akt signaling pathway is elevated, which can activate APP. APP can be metabolized through amyloid and non-amyloid pathways. The amyloid pathway is the secondary APP metabolic pathway. The neurotoxic Aβ is produced when the gene is mutated or under oxidative stress by the cleavage of key enzymes such as ACE-1 and γ-secretase. This causes Aβ deposition to form amyloid plaques [40]. When Aβ deposition can not be metabolized properly, it can only directly induce neuronal apoptosis by increasing the activity of apoptosis protease and enhancing the aggregation of intracellular free radicals. Aβ deposition can also reduce the transmission of glutamate neurons and cause synaptic loss, thereby indirectly impairing memory and cognitive functions. Therefore, we inferred that miRNA-320 might cause Aβ deposits by altering *IGF-1* mRNA and APP protein, leading to the occurrence of POD.

Nevertheless, the present study had four limitations. Firstly, the number of animal study was limited. Sample sizes should be increased in future studys. Secondly, this study monitored the progression of the disease by measuring the changes in miRNA-320 level in the peripheral blood through large-scale clinical studies. Thirdly, we will measure the changes in the expression levels of other miRNAs in peripheral blood to provide a theoretical basis to clarify the postoperative mechanisms underlying cognitive dysfunction through a large-scale clinical study. Finally, we will measure the changes in miRNA-320 level between Caucasians and Han Chinese in future studys.

## Conclusion

The miRNA-320 may promote the occurrence of POD by up-regulating *IGF-1* mRNA and APP protein. This research provides a new target for the diagnosis and treatment of postoperative POD.

## Supplementary Information

**Additional file 1.**

## Data Availability

The datasets used and/or analyzed during the current study are available from the corresponding author on reasonable request.
