# Supplementary figures and images for: Correlation between microRNA-320 and postoperative delirium in patients undergoing tibial fracture internal fixation surgery
Source: BMC Anesthesiol. 2022 Mar 22;22:75. doi: 10.1186/s12871-022-01612-w (PMC8939177; doi:10.1186/s12871-022-01612-w)

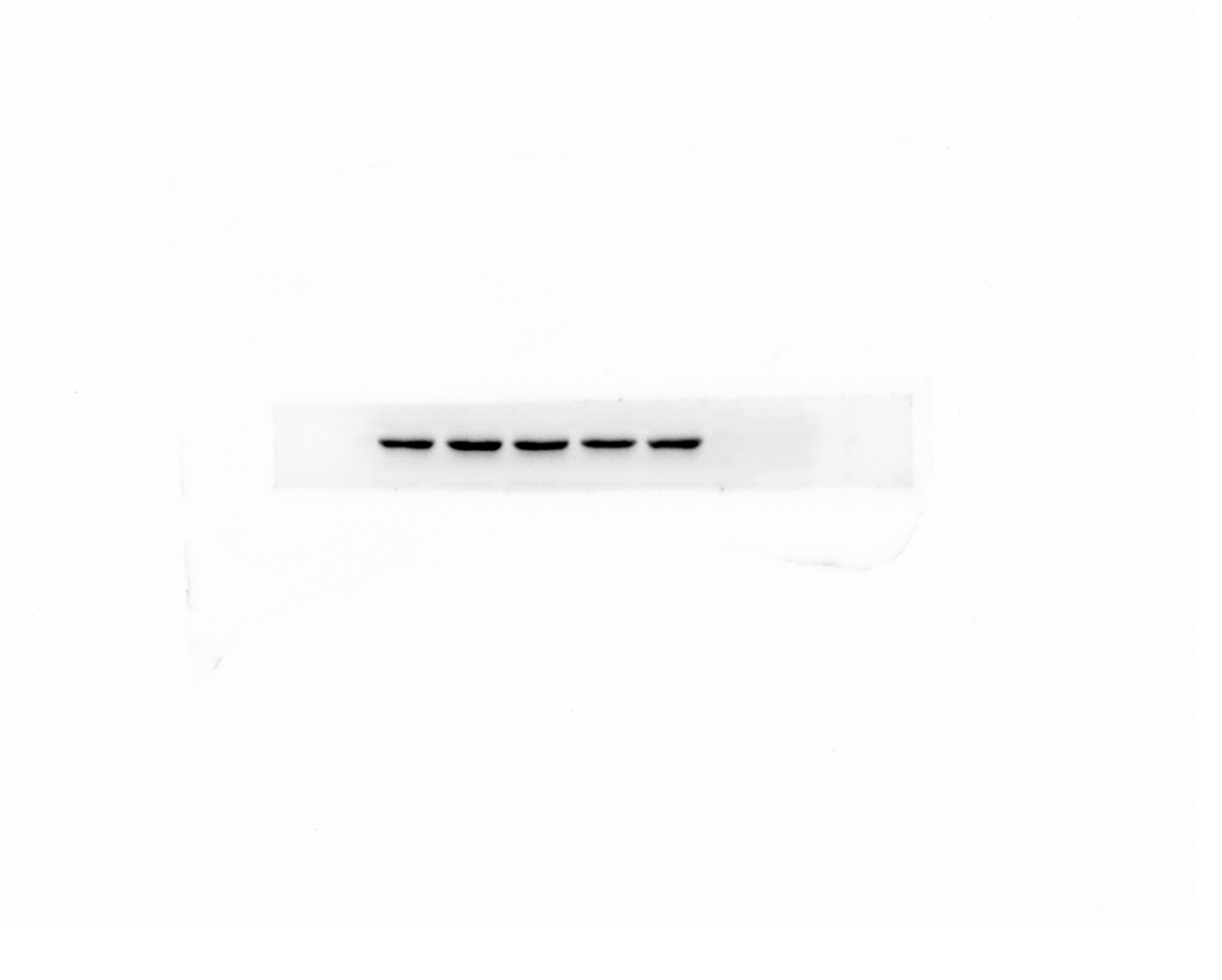

Supplement: Supplementary file 1 — Additional file 1. [file 12871_2022_1612_MOESM1_ESM.zip › actin--1d.jpg]

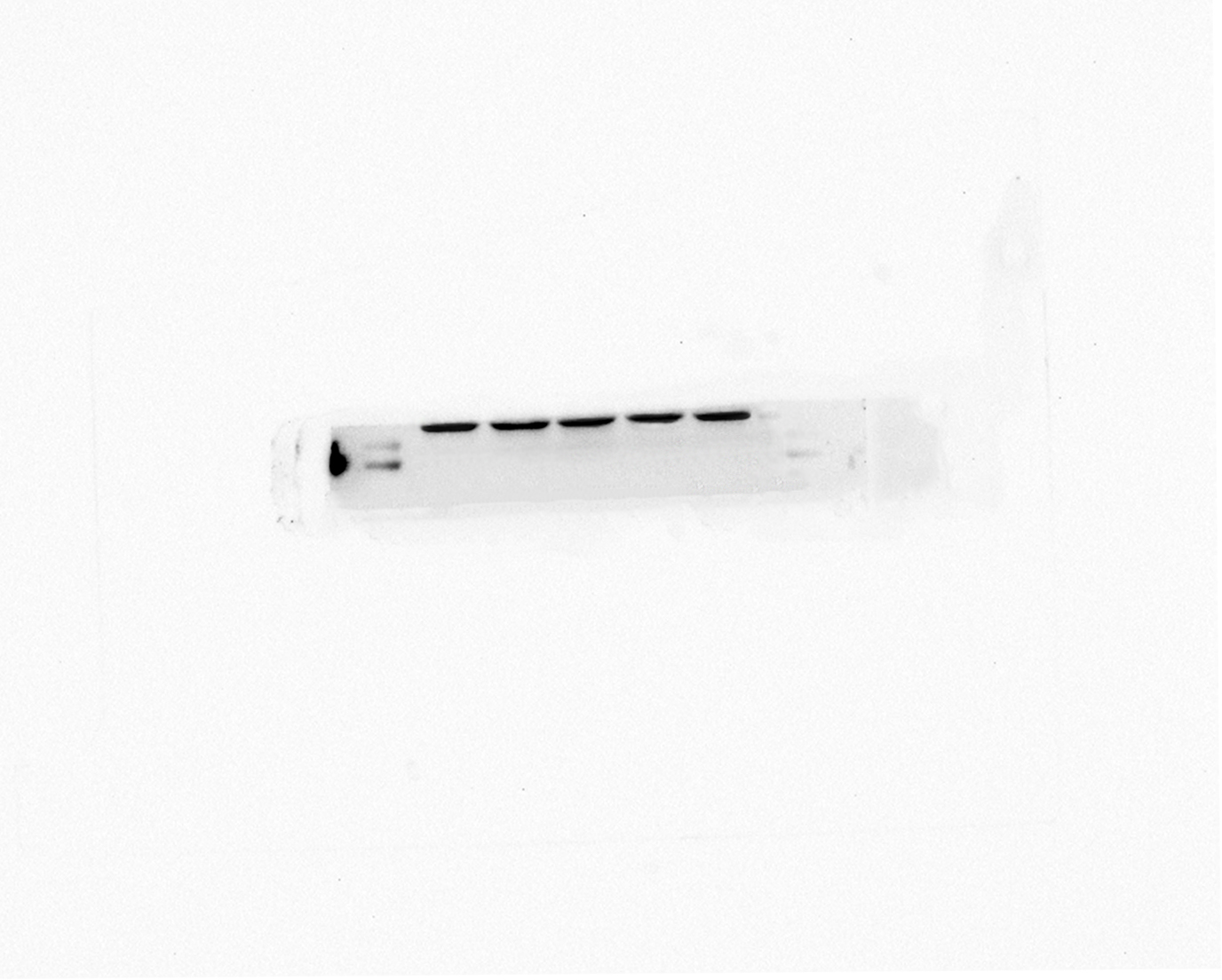

Supplement: Supplementary file 1 — Additional file 1. [file 12871_2022_1612_MOESM1_ESM.zip › actin--3d.jpg]

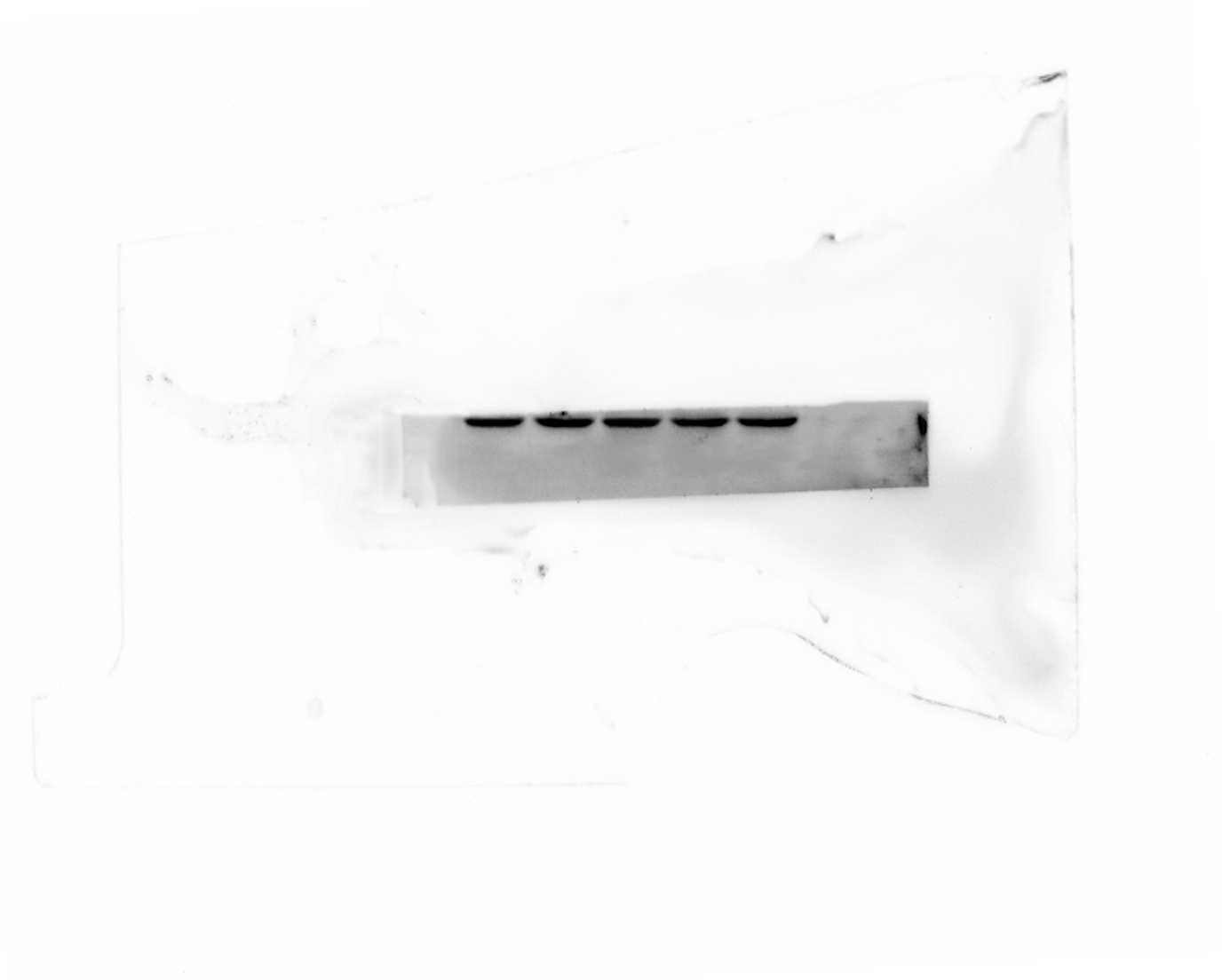

Supplement: Supplementary file 1 — Additional file 1. [file 12871_2022_1612_MOESM1_ESM.zip › actin--7d.jpg]

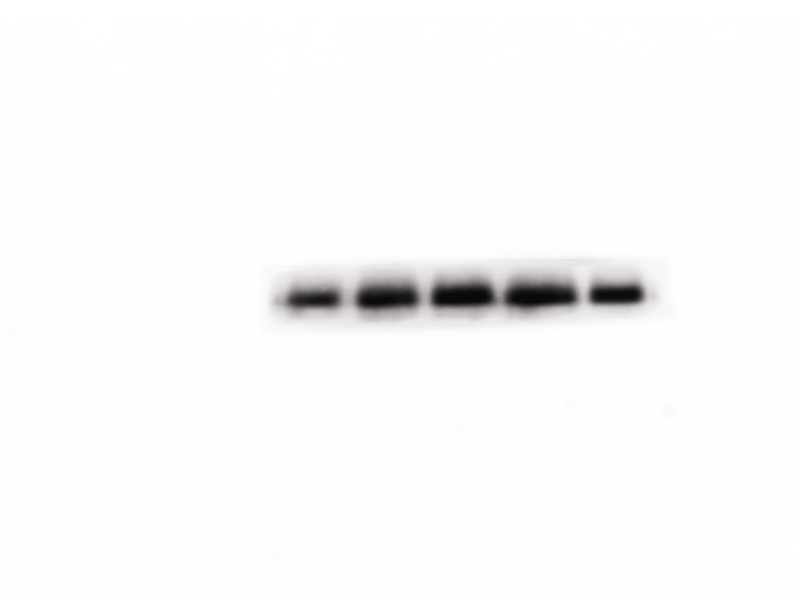

Supplement: Supplementary file 1 — Additional file 1. [file 12871_2022_1612_MOESM1_ESM.zip › App--1d.jpg]

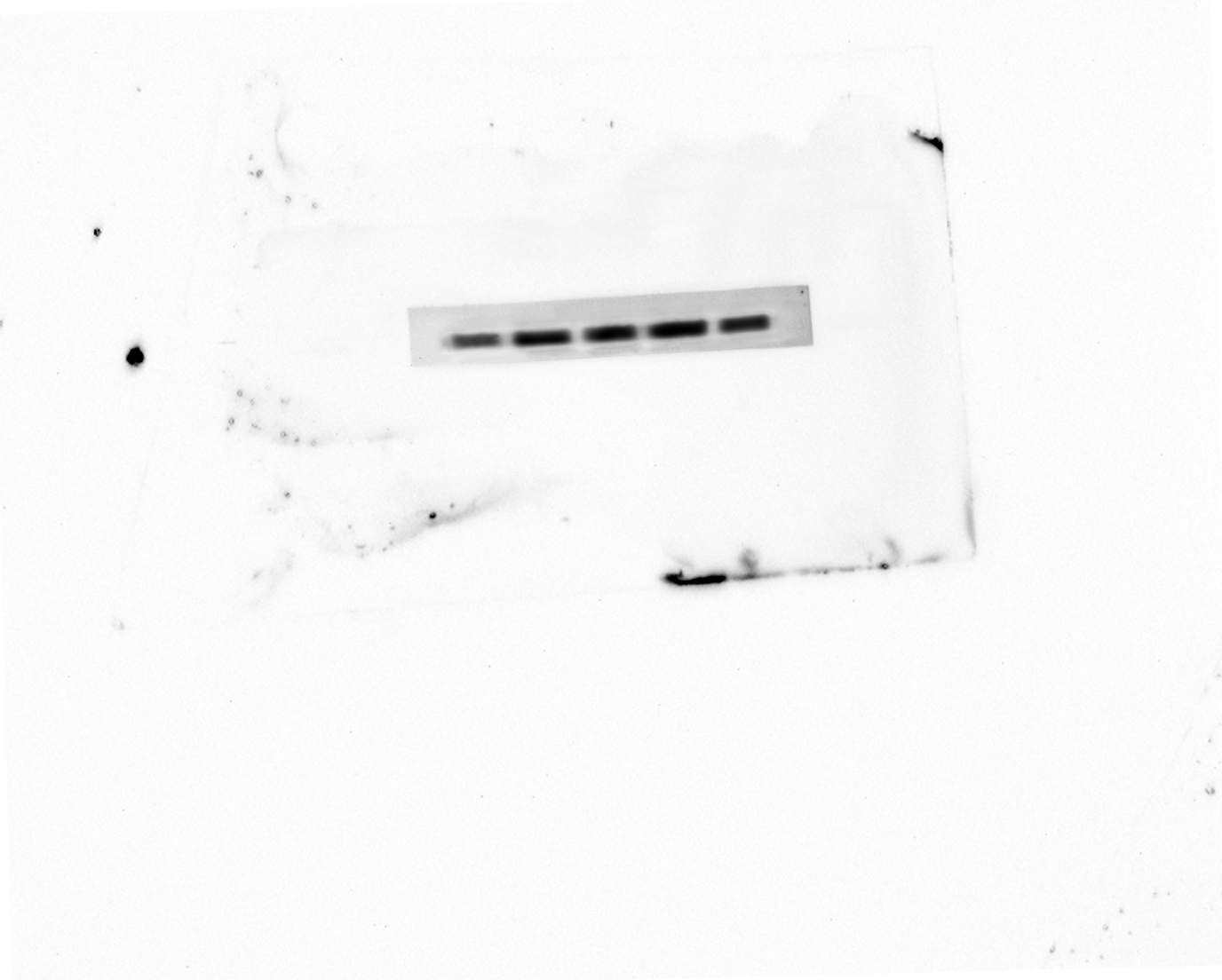

Supplement: Supplementary file 1 — Additional file 1. [file 12871_2022_1612_MOESM1_ESM.zip › App--3d.jpg]

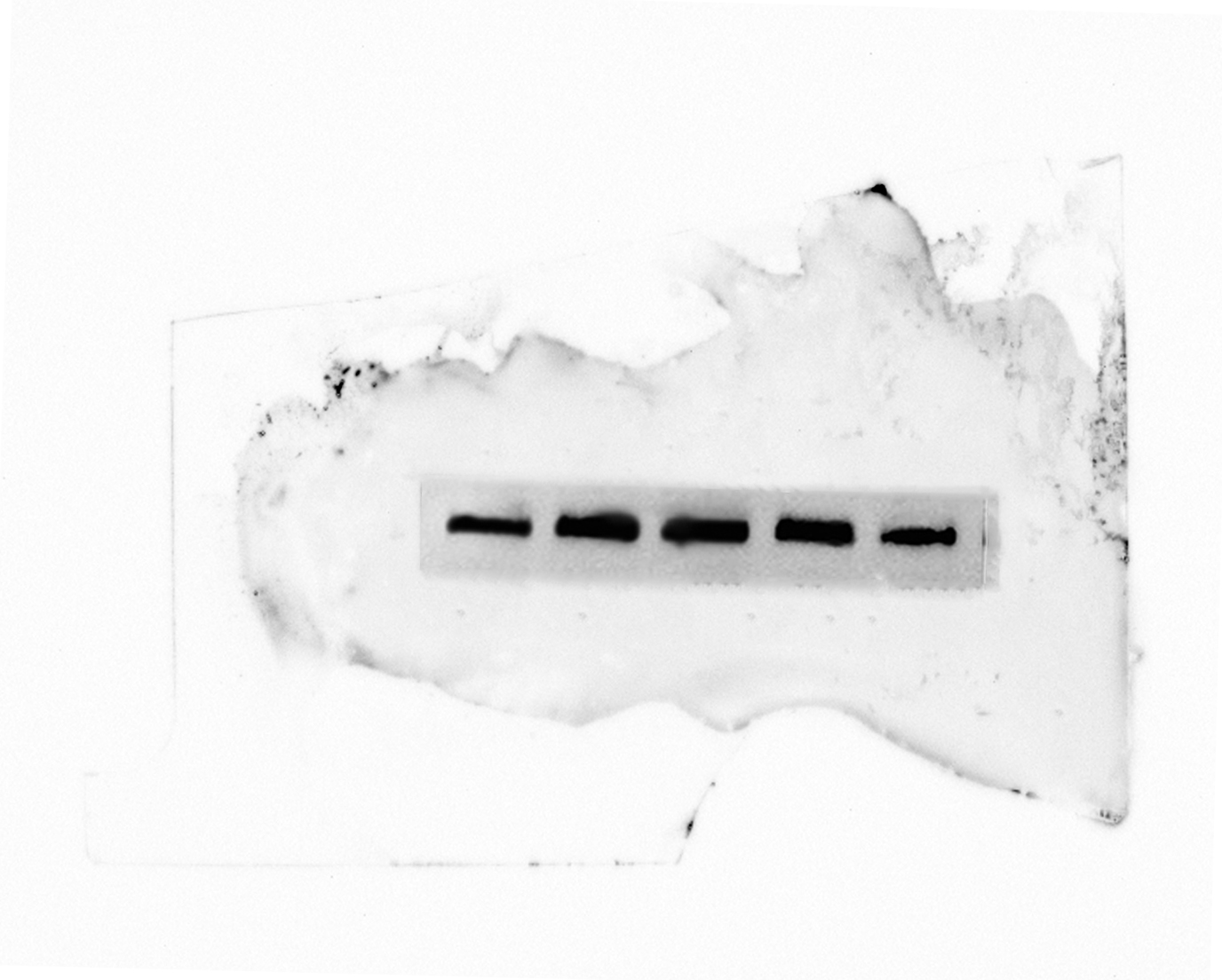

Supplement: Supplementary file 1 — Additional file 1. [file 12871_2022_1612_MOESM1_ESM.zip › App--7d.jpg]

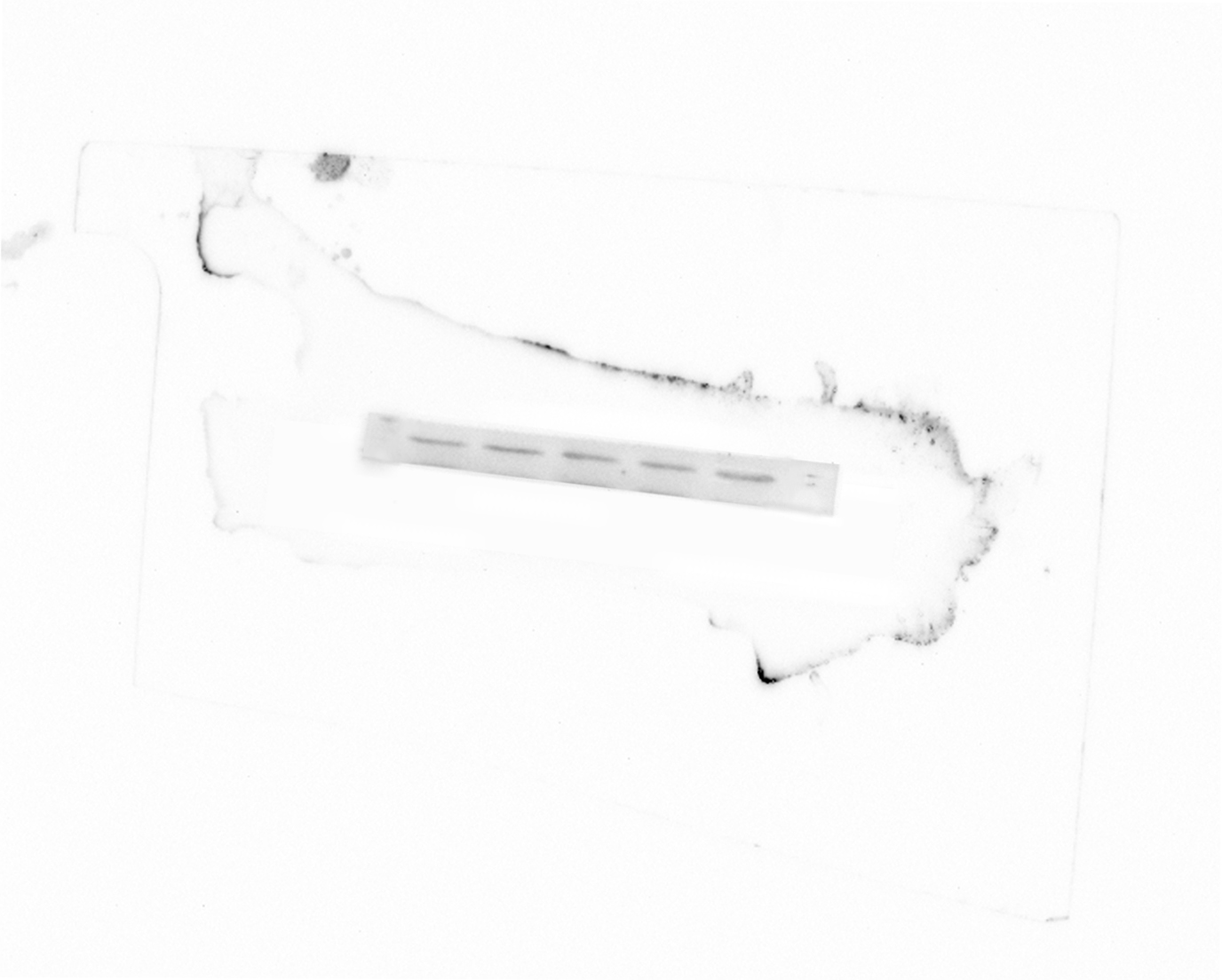

Supplement: Supplementary file 1 — Additional file 1. [file 12871_2022_1612_MOESM1_ESM.zip › IGF1--1d.jpg]

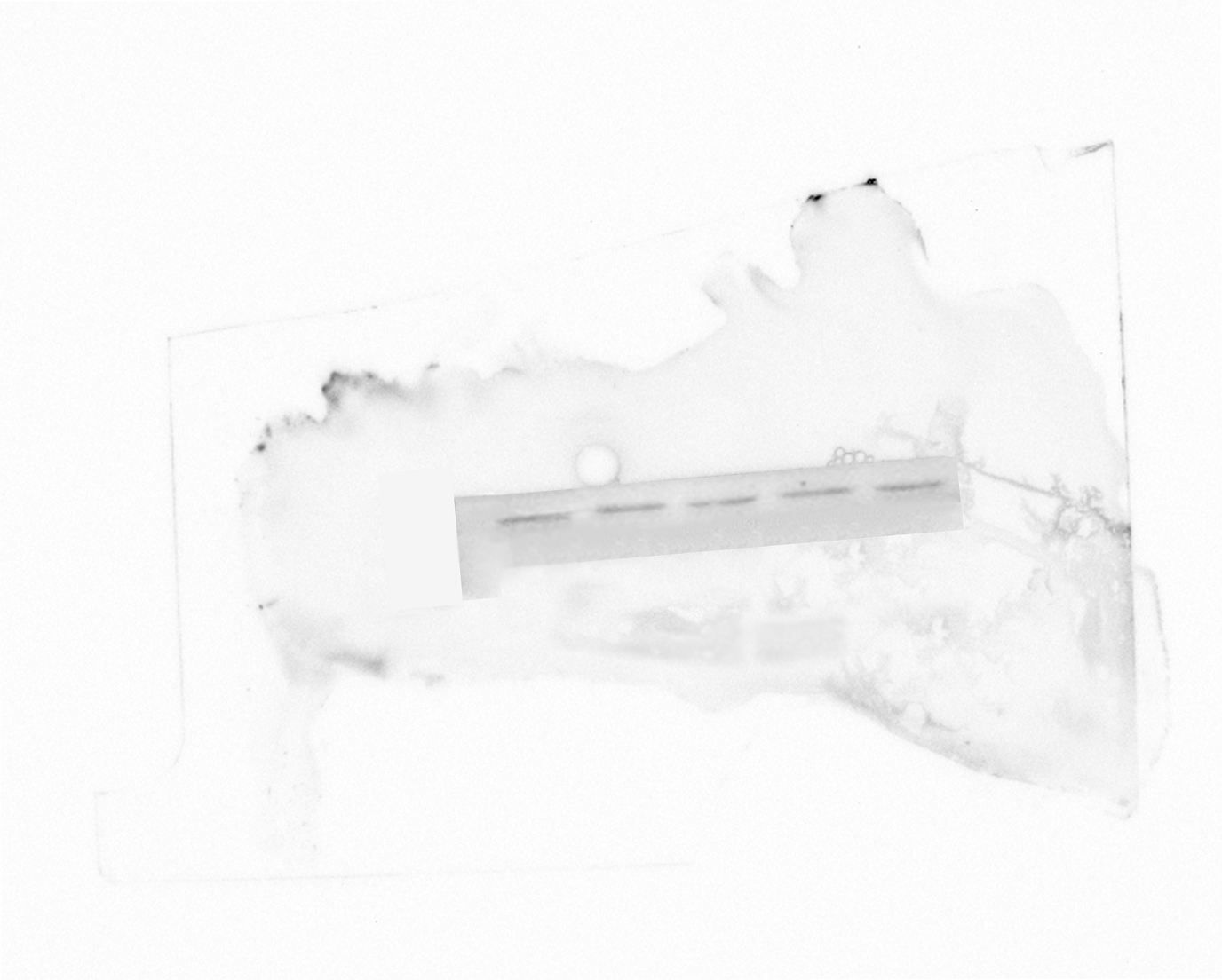

Supplement: Supplementary file 1 — Additional file 1. [file 12871_2022_1612_MOESM1_ESM.zip › igf-1---3d.jpg]

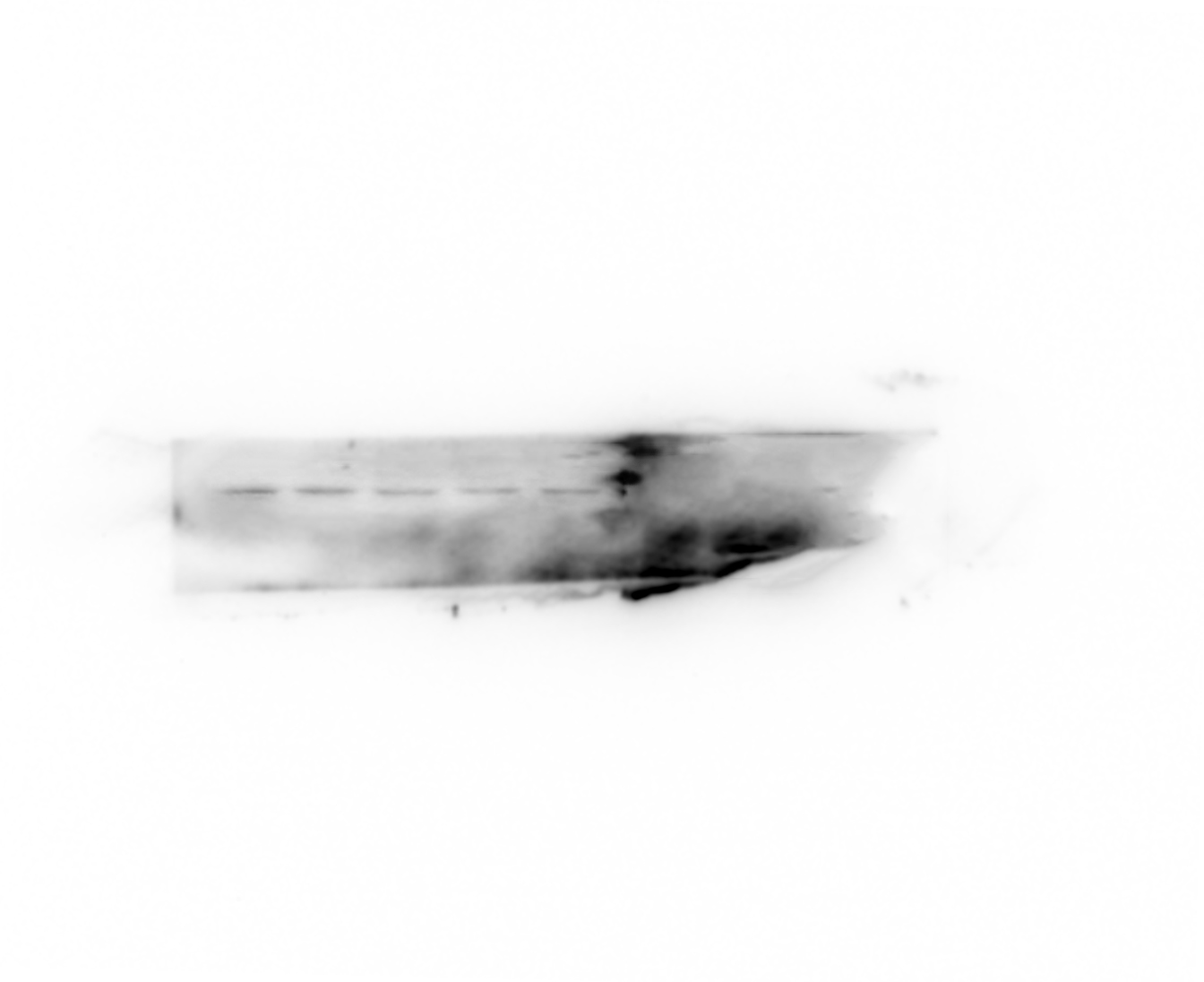

Supplement: Supplementary file 1 — Additional file 1. [file 12871_2022_1612_MOESM1_ESM.zip › igf-1---7d.jpg]
